# Preparation and Modification of Polydopamine Boron Nitride—Titanium Dioxide Nanohybrid Particles Incorporated into Zinc Phosphating Bath to Enhance Corrosion Performance of Zinc Phosphate-Silane Coated Q235 Steel

**DOI:** 10.3390/ma16103835

**Published:** 2023-05-19

**Authors:** Mustafa Muhammad, Ruina Ma, An Du, Yongzhe Fan, Xue Zhao, Xiaoming Cao

**Affiliations:** Key Laboratory for New Type of Functional Materials in Hebei Province, Tianjin Key Laboratory Material Laminating Fabrication and Interface, Tianjin Engineering and Technology Center for Environmental-Friendly Coating on Pipeline, School of Materials Science and Engineering, Hebei University of Technology, Tianjin 300132, China; maryna@126.com (R.M.); duan@hebut.edu.cn (A.D.); zhaoxue@hebut.edu.cn (X.Z.)

**Keywords:** zinc-phosphating bath, corrosion-resistance, steel, dopamine, boron nitride, organosilane coating, nano-TiO_2_

## Abstract

In this work, PDA@BN-TiO_2_ nanohybrid particles were incorporated chemically into a zinc-phosphating solution to form a robust, low-temperature phosphate-silane coating on Q235 steel specimens. The morphology and surface modification of the coating was characterized by X-Ray Diffraction (XRD), X-ray Spectroscopy (XPS), Fourier-transform infrared spectroscopy (FT-IR), and Scanning electron microscopy (SEM). Results demonstrate that the incorporation of PDA@BN-TiO_2_ nanohybrids produced a higher number of nucleation sites and reduced grain size with a denser, more robust, and more corrosion-resistant phosphate coating compared to pure coating. The coating weight results showed that the PBT-0.3 sample achieved the densest and most uniform coating (38.2 g/m^2^). The potentiodynamic polarization results showed that the PDA@BN-TiO_2_ nanohybrid particles increased phosphate-silane films’ homogeneity and anti-corrosive capabilities. The 0.3 g/L sample exhibits the best performance with an electric current density of 1.95 × 10^−5^ A/cm^2^, an order of magnitude lower than that of the pure coatings. Electrochemical impedance spectroscopy revealed that PDA@BN-TiO_2_ nanohybrids provided the greatest corrosion resistance compared to pure coatings. The corrosion time for copper sulfate in samples containing PDA@BN/TiO_2_ prolonged to 285 s, a significantly higher amount of time than the corrosion time found in pure samples.

## 1. Introduction

Surface pretreatment is often used in industrial processing as a conventional technique for treating zinc layers to protect and improve their corrosion performance. The substrate’s surface is treated in a specialized way to create a protective layer that connects the organic coating and the metal beneath, conceding immediate lubrication and offering immunity against corrosion [1]. Recent studies revealed that chrome coating demonstrated good chemical conversion capabilities compared to chromate, molybdate, and phosphate coatings. However, due to the environmentally hazardous nature of chromium salts, phosphate coatings emerged as the most practical option for surface preparation with the most negligible adverse impact on the environment [2,3,4].

Phosphating has become an eco-friendly technique used in both the automotive and domestic industries with the advancement of modern industry [1] to improve the surface treatment of metals and enhance their corrosion resistance [5]. Over the years, applying various catalysts to obtain effective phosphate coatings at elevated temperatures has adversely impacted the environment and human health [6]. Although zinc phosphate coating has some limitations, including lower corrosion resistance than desired, it is often still employed in industrial settings as its performance impacts overall coating efficacy in terms of corrosion resistance [7].

Two-dimensional nanomaterials, such as graphene and hexagonal boron nitride (h-BN), are gaining increasing attention in the coating industry due to their potential to enhance coating performance significantly. The application of two-dimensional nanomaterials as corrosion-inhibiting coatings has been the focus of considerable investigation in recent years. Their small size and high surface area to volume ratio provides them with unique chemical and physical properties that can be harnessed to improve the performance of corrosion-resistant coating [8,9]. Studies have shown that even minimal changes to nanomaterial’s chemical or physical structure can considerably impact their corrosion resistance capability. The high aspect ratio of 2D nanomaterials also enables their easy diffusion into the coating lattice, which further helps to improve their overall efficiency and durability [3,10].

Additionally, their excellent binding ability with other materials improves coatings’ adhesion, wear, and resistance properties [3,10,11].

Using two-dimensional (2D) nanomaterials for phosphating has been developed as a promising technique due to the nanosheet’s increased surface area, which can facilitate more efficient bonding between phosphate molecules and the substrate surface. The larger surface area provided by nanosheets enables them to bind phosphate molecules, while their remarkable chemical stability makes them an ideal supplementary protective barrier against harsh elements. The incorporation of graphene oxide (GO) and boron nitride nanosheets (BNs) into a phosphating solution was investigated by Xie et al. [7,8], as these materials possess a high specific surface area that can improve the corrosion resistance of the coating. Similarly, h-BN was introduced by Cui et al. into epoxy resin, yielding an enhancement of corrosion protection due to its ability to interrupt crack propagation and decrease coating porosity, resulting in less permeation of the corrosive medium [12]. Furthermore, due to their chemical stability, materials such as graphene oxide (GO) and boron nitride (BN) can be supplementary barriers when exposed to corrosive mediums.

Additionally, 2D nanomaterials have given further control over the phosphating process, making possible a more even distribution of the coating over the substrate. However, the synthesis of these nanomaterials has posed a variety of challenges [7,13]. However, nanoparticles could improve organic coatings’ mechanical and anti-corrosive properties due to their small size and form, even at low concentrations. One of the most challenging research areas is ensuring a functional interface between nanoparticles and their polymer host resin and ways to strengthen their interaction [14]. Using nanoparticles modified with nanomaterials such as boron nitride (BN) and graphene oxide (GO) for phosphating could potentially improve the quality of coatings in the industrial sector. Modifying the composition of coating materials used in this sector may provide benefits, such as better adhesion and greater longevity [15].

An essential metal oxide, Titanium dioxide (TiO_2_), has proven incredibly beneficial in recent years due to its non-toxicity, affordability, and excellent light, thermal, and other properties. It has also been applied to many fields, such as water and air purification, surface self-cleaning, self-sterilization, and photoelectric devices [16]. Shibli et al. reported that the phosphate coatings developed in the presence of nano TiO_2_ on the galvanized mild steel at 55 °C resulted in large crystals, greater surface coverage, and enhanced barrier protection characteristics [17]. Inorganic nanoparticles, such as silica (SiO_2_), titania (TiO_2_), and zinc oxide (ZnO), are being increasingly studied recently to optimize the number of nucleation sites and boost the efficiency of eco-friendly phosphate coatings [18]. Yu et al. discovered that nanosized TiO_2_ coated onto the surface of GO created a TiO_2_-GO composite material capable of enhancing the corrosion resistance of epoxy resin when dispersed in the resin coating to increase the compatibility and macroscopic properties of the GO-epoxy composite [19]. In addition, Shuli Wang et al. also studied the effectiveness of using nano-PDA@GO-TiO_2_ composites as nanofillers to reinforce the physical properties and corrosion resistance of waterborne epoxy coatings [20]. In terms of corrosion protection, these particles can act as catalysts for chemical reactions, accelerating the process and boosting their protective capabilities. In particular, TiO_2_ nanoparticles have proved especially useful in zinc phosphate coatings due to their high corrosion stability and non-toxicity. This active ceramic material can increase the crystal nucleation rate and form a finer coating structure, improving coating performance [11,17].

Polydopamine, an eco-friendly reducing agent, can be easily self-polymerized and bonded onto the surfaces of various organic and inorganic materials, forming a polydopamine layer. It also features various functional groups, such as catechol, amines, and imines, that can be used for the covalent attachment of desired molecules [19,20,21]. Polydopamine (PDA) has been proposed as a potential choice to alter the hexagonal boron nitride (h-BN) nanosheet’s surface to yield a highly active and multifunctional surface that caters to a range of applications. Dopamine (DA) efficiently eliminates the labile oxygen groups present in GO and consolidates the formation of polydopamine-functionalized graphene oxide (PDA-GO). The oxidation of DA results in rearranging and intermolecular cross-linking reactions, which improves the adhesion between GO and epoxy, allowing a homogenous dispersion of GO within the epoxy matrix. As a result, the epoxy composites’ thermal conductivity and mechanical properties were enhanced [20]. The low corrosion resistance of phosphate coatings can be attributed to micropores in the coating layer, which can be addressed by integrating other environmentally friendly organo-silanes, such as KH560, to enhance corrosion resistance [1]. Due to the sealing effect of silane (KH560), zinc phosphate coatings exhibited reduced defect levels upon employing silane (KH560), yielding a composite coating with improved corrosion resistance [1,21]. Muhammad et al. successfully developed a phosphate-silane layer on Q235 steel, incorporating polydopamine-modified boron nitride (BNs) nanosheets into the zinc phosphating bath with excellent coating weight and corrosion resistance [1].

In this study, we have synthesized an environmentally friendly phosphate-silane coating on the Q235 steel by introducing polydopamine (PDA) modified BN/TiO_2_ (PBT) nanohybrid particles into the phosphating bath. A simple, straightforward technique was employed to chemically join the BN/TiO_2_ nanohybrid particles and zinc phosphate coatings through silane (KH560) by the surface modification of BN/TiO_2_ nanohybrid particles modified by polydopamine (PDA). The coating morphology was characterized by X-ray diffraction (XRD, X-ray photoelectron spectroscopy (XPS), Scanning Electron Microscope (SEM), Fourier-transform infrared spectroscopy (FT-IR), and electrochemical and mass measurements.

## 2. Materials and Methods

### 2.1. Materials

Shanghai Macklin Biochemical Co., Ltd. (Shanghai, China) supplied Phosphoric acid 85%, Phosphorous acid 99%, and epoxy functionalized silane (KH560). Tianjin Aladdin Co., Ltd. provided Zn(H_2_PO_4_)_2_·2H_2_O, Zn (NO_3_)_2_·6H_2_O, and NaOH, nano-Titanium dioxide (TiO_2_), respectively. Hexagonal Boron Nitride (BNs) nanosheets, polydopamine hydrochloride (PDA), and Tris (hydroxymethyl aminomethane) were acquired from Shanghai Aladdin Biochemical Technology Co., Ltd. (Shanghai, China). Specimens of grade Q235 steel with the composition listed in Table 1 were cut into 40 mm × 40 mm × 3 mm pieces and used in the zinc phosphating experiments. This study utilized chemicals at an empirical grade without further refinement or purification.

### 2.2. Preparation of PDA@BN/TiO_2_ (PBT) Nanohybrids

**Scheme 1:**
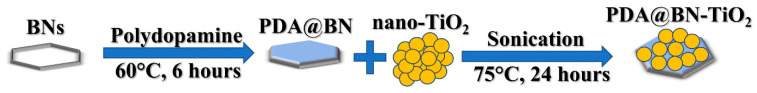
The preparation procedure of Polydopamine modified Boron Nitride/Titanium dioxide (PDA@BN/TiO_2_).

The synthesis of h-BN@PDA was prepared following our previous literature [1] and was optimized based on them. The preparation of PDA@BN/TiO_2_ (PBT) Nanosheets is shown in Scheme 1. Firstly, A mixture of 0.6 g two-dimensional boron nitride nanosheets, 0.16 g Tris hydroxyethyl aminomethane, ethanol (30 mL), and 0.24 g dopamine hydrochloride was sonicated for 30 min and stirred at 60 °C for 6 h. This was performed to ensure the nanosheet dispersion, prevent poly (dopamine) polymerization, and accumulate the boron nitride nanosheets during the reaction process. It was then added 0.30 g nano-TiO_2_ particles into the dispersion. The mixed solution was continuously stirred and heated for 30 min at 75 °C for 24 h, while the color of the solution changed from yellowish brown to black. After the reaction, the hybrid system was centrifuged. The obtained product was frequently washed with ethanol and pure water several times to remove unreacted dopamine and other reagents and freeze-dried under a vacuum to a constant mass to obtain the final product, nano-PDA@BN-TiO_2_ (PBT) nanohybrid particles.

### 2.3. Pre-Preparation of Mild Steel Sheets

The Q235 steel specimens were given an initial treatment of grinding with 600 and 800-grit sandpaper for dust removal or impurities on their surface. The rust was then removed during the next step of polishing. After that, they were degreased in a 10.0 wt.% solution of NaOH heated to 40 °C for 10 min. The specimens were washed in deionized water and air-dried at room temperature.

### 2.4. Phosphating Procedure

Table 2 outlines the details of the zinc phosphating bath solution, including the bath’s chemical components and corresponding operating conditions. The phosphating bath comprised 85% phosphoric acid, 99% phosphorous acid, KH560 silane, Zn(H_2_PO_4_)_2_·2H_2_O, PDA@BN-TiO_2_ nanohybrid particles with concentrations from 0 g/L to 1.2 g/L and Zn(NO_3_)_2_·6H_2_O dissolved into 1 L of distilled water. Steel substrates were immersed in the zinc-phosphating bath at 40 °C for half an hour, followed by ultrasonication with continuous stirring for 30 min. After immersion, specimens were rinsed with pure water to remove any lingering acid or salt that could have been absorbed. The steel specimens were dried with compressed air. Afterward, the specimens were subjected to further characterization.

### 2.5. Characterization of PDA@BN/TiO_2_

Using scanning electron microscopy (SEM) (JSM-06510A, JEOL, Japan), the characteristics of phosphate-silane coating on steel samples were examined powered by an acceleration voltage of 20 kV, X-ray Photoemission Spectroscopy (XPS) (ESCALAB 250Xi, Thermo Scientific) and X-ray Diffraction (Bruker D8 ADVANCE).

The mass per unit area (MPA) of phosphate silane coatings was calculated to determine their coating weight using the equation $MPA=\frac{\left(m_{1}-m_{2}\right)}{S}$ “m_1_” and “m_2_” refer to steel sample mass discarded after both pretreatment and post-treatment phosphating, and S stands for the phosphate steel specimens’ surface area [22]. A particular solution was employed to completely dissolve the phosphate coating on the steel specimens to calculate the thickness of the coating. After immersing the steel specimens in the solution at a temperature of 70 °C for 5 min, the bare steel was revealed. Subsequently, the mass of the steel specimens before and after the process was measured and labeled m_1_ and m_2,_ respectively [23].

### 2.6. EIS Measurements

The electrochemical tests for the anti-corrosive properties of the phosphate-silane coating of steel were carried out using Potentiodynamic polarization (Tafel) and Electrochemical impedance spectroscopy (EIS). An Ag/AgCl reference electrode was conducted in a conventional three-electrode setup with a platinum (Pt) counter electrode and the steel specimens as the working electrode with an exposed area of 1 cm^2^. The experiments were conducted at room temperature with a 3.5% NaCl aqueous electrolyte in a CHI660E electrochemical workstation (Chenua, Shanghai, China). The EIS measurements were conducted with a 400 s stabilization time, with a 5 mV amplitude between a 10 mHz–100 kHz frequency range, recordable with ZSimp Win (EChem Software version 3.60, Q5 Ann Arbor, MI, USA). The Tafel extrapolation was performed by plotting the cathodic to anodic polarization curve (OCP − 300 mV–OCP + 300 mV) at a scan rate of 1 mV/s to access the corrosion current density, electrochemical parameters, and corrosion potential. All measurements were repeated 3 times for accuracy, with the average value adopted in the analysis.

## 3. Results

### 3.1. Characterization of PDA@BN/TIO_2_ (PBT)

#### 3.1.1. SEM Analysis

**Figure 1:**
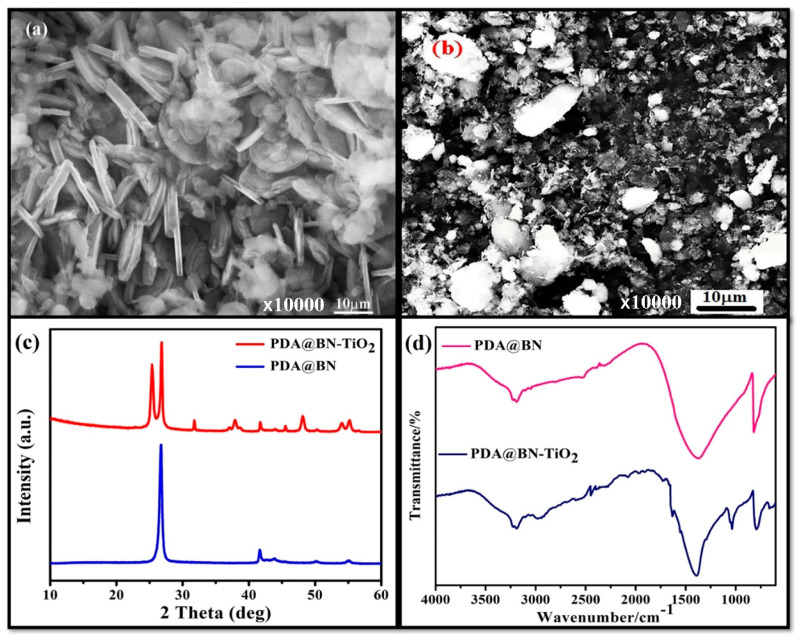
SEM images of (**a**) PDA@BN, (**b**) PDA@BN-TiO_2_, and (**c**) XRD patterns of PDA@BN, and PDA@BN-TiO_2_. (**d**) FT-IR spectra of PDA@BN and PDA@BN-TiO_2_.

Figure 1a,b show the SEM pictures of the polydopamine-modified BNs nanosheets and PDA@BN-TiO_2_ (PBT) nanohybrid particles, respectively. Boron Nitride (BN) nanosheets generally possess a commercially round disk form, displaying strong Van der Waals interactions and being packed and gathered together to form nanosheets of 256 nm in diameter and 34 nm thick [24]. The modification of BN nanosheets results in the disappearance of particle aggregation, while tiny and grainy particles emerge on the surface, forming poly (dopamine) particles due to the modification process (see Figure 1a). Figure 1b shows that h-BN nanosheets have numerous protuberances affixed to their surfaces, which are believed to be TiO_2_ nanoparticles self-assembled on the outer layer of PDA@BN. It is evident from Figure 1b that the PDA@BN nanosheets have been proven to be effective in preventing aggregations of TiO_2_ nanoparticles and also have good dispersibility on the surface of PDA@BN. The PDA@BN-TiO_2_ nanohybrid particles demonstrate a prototypical core-shell structure in which the h-BN nanosheet takes up the role of the core and the PDA organic layer and adheres to TiO_2_ particles that constitute the shells.

#### 3.1.2. XRD Patterns

The XRD spectrum in Figure 1c gives information regarding the phase patterns of boron nitride and TiO_2_, respectively. PDA@BN nanosheets have prominent peaks at 26.9°, 41.6°, 43.9°, 50.2°, and 55.1°, which corresponds to (002), (100), (101), (102), and (004) planes, respectively [1,25]. The characteristic peaks of TiO_2_ appeared on PDA@BN-TiO_2_ particles, with the lattice planes of the anatase phase of (101) = 25.31°, (112) = 38.01°, (200) = 48.02°, (105) = 54.45°, and (204) = 62.59° theta values (JCPDS card no. 21-1272), respectively, also demonstrates the successful modification due to PDA layer [26,27]. However, after the polydopamine (PDA) modification, a distinct diffraction peak in the 22–38° was identified in the resulting nano- PDA@BN-TiO_2_ hybrids. It indicates the successful incorporation of Poly (dopamine) (PDA) on the BN nanosheets, which causes the stacking of the benzene rings in the dopamine (DA) molecules [20,28].

#### 3.1.3. FT-IR Spectroscopy

Figure 1d illustrates the FT-IR spectra of PDA@BN nanosheets and PDA@BN-TiO_2_ nanohybrid particles. The characteristic peaks corresponding to the –OH and –CH2– stretching modes at 3442 cm^−1^ and 2854 cm^−1^ belonging to poly (dopamine) were observed on the surface of h-BN particles. The clear peaks near 1376 cm^−1^ signify vibrations of B-N stretching, and B-N-B bending vibrations are indicated by the peaks close to 819 cm^−1^. The symmetric and asymmetric –CH_2_– stretching modes are indicated by the PDA@BN and small absorption bands at 2924 cm^−1^ and 2854 cm^−1^, respectively. The range of C–C bending vibrations in phenol rings is between 1300 cm^−1^ and 1600 cm^−1^ [29,30]. When TiO_2_ was added to the PDA@BN, the FT-IR curve of the nano-PDA@BN- TiO_2_ hybrid particle showed a wide peak at 562 cm^−1^ and 1115 cm^−1^, which were attributed to the Ti–O–Ti and Ti–O vibration peak in TiO_2_ particle [20,27,31,32]. In addition, a new characteristic peak at 1055 cm^−1^ corresponds to the C–N stretching originating from PDA [20]. The peaks corresponding to 3400 cm^−1^ and 1631 cm^−1^ were indexed to hydroxyl groups’ stretching and bending vibration, respectively. The hydroxyl groups with a negative charge on the h-BN nanosheets and the oxygen vacancies in TiO_2_ nanoparticles could lead to a tight contact between h-BN sheets and TiO_2_ nanoparticles. The large and bulky size of boron nitride reduces its dispersion stability. It creates difficulties for application in anti-corrosion processes. FT-IR data confirmed the successful coating of polydopamine onto the BN-TiO_2_ nanohybrid particles, as evidenced by –OH and –CH_2_– stretching modes at 3442 cm^−1^ and 2854 cm^−1^, respectively. 

#### 3.1.4. Digital Photos Dispersibility

**Figure 2:**
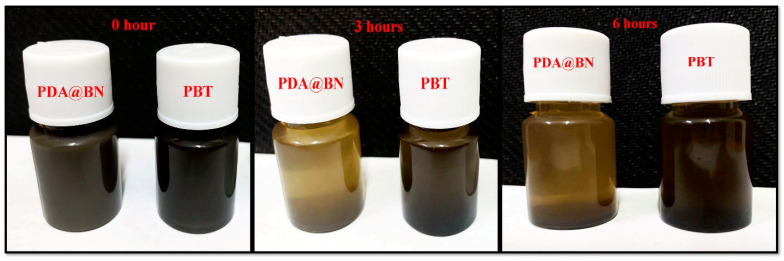
Dispersion stability in a phosphating bath as captured by digital photography of PDA@BN and PDA@BN-TiO_2_ nanohybrids at 0 h, 3 h, and 6 h.

Due to the difficulty of altering the chemical inertia of BN/TiO_2_ nanohybrid particles, polydopamine (PDA) is employed for their surface modification, as demonstrated in Figure 2. Figure 2 presents images of h-BN@PDA nanosheets and PDA@BN/TiO_2_ nanohybrid particles in the zinc-phosphating bath, with a 0.3 g/L concentration after ultra-sonication for 5 min, exhibiting the greyish color, respectively.

However, polydopamine @ boron nitride nanosheets were set at the base, while the PDA@BN-TiO_2_ nanohybrid particles remained suspended in the zinc-phosphating solution after three and six hours, respectively. As a result of the PDA layer, PDA@BN-TiO_2_ nanohybrid particles lowered their ability to interact with the zinc-phosphating solution as time passed and confirmed the polydopamine modification of BN/TiO_2_ nanohybrid particles (Figure 2).

### 3.2. FESEM of PDA@BN/TiO_2_ Incorporated Phosphate-Silane-Coated Samples

**Figure 3:**
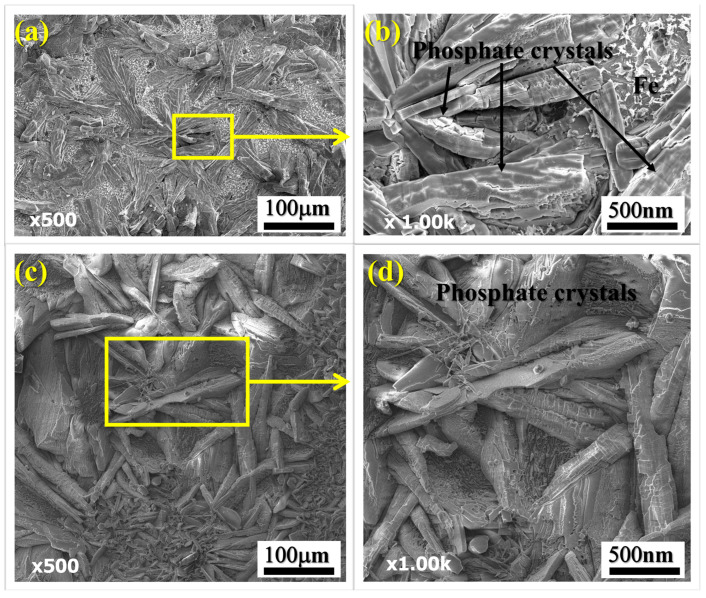
High-resolution SEM of phosphate coating in PBT-0 (**a**,**b**) and PBT-0.3 (**c**,**d**).

High-quality SEM was employed to study the PBT-0 (pure coated) and PBT-0.30 g/L (coated with PDA@BN/TiO_2_) phosphate-silane-coated samples to demonstrate the existence of PDA@BN-TiO_2_ nanohybrid particles into the phosphate-silane film. The phosphate coating formation is a complicated and complex electrochemical procedure. Figure 3 illustrates uniformity and compactness in both samples, achieving a smoother surface in the PBT-0.30 g/L sample. In the PBT-0 sample, large phosphate crystals were present on the surface, and parts were left uncoated, as shown in Figure 3a. The PBT-0.30 g/L showed an even distribution of the PDA@BN-TiO_2_ nanohybrid particles over the surface and a highly compressed phosphate presence, as shown in Figure 3b.

Despite this, during the phosphating process, the phosphate group is transferred from the primary to the tertiary form, which is insoluble, settles on the metal’s surface, and starts complex chemical and electrochemical reactions to form the phosphate coating. Ghali et al. investigated five steps involved in developing conversion coatings on steel, which included exposing the metal to an electrochemical reaction in an acid, reintroducing a small amount of phosphoric acid (H_3_PO_4_) to the steel, forming an uneven film, irregular crystallization, and reforming crystalline structures [33].

An ordinary phosphating bath such as PBT-0 does not include any substances that can spur the progress of formation into crystals Figure 3a. However, Figure 3c reveals that stacked phosphate crystals exist and are much more uniform in PBT-0.30 specimens than in PBT-0 specimens. The analysis of the FESEM reveals that the final phosphate coating was efficiently improved by incorporating PDA@BN-TiO_2_ into the phosphating bath, which leads to the compactness of the phosphate crystals attributed to the coalescence of phosphate crystals. Additionally, it is worth noting that the presence of PDA@BN-TiO_2_ improves the interfacial adhesion of the phosphate-silane coating, as seen in Figure 3d.

### 3.3. EDS mapping of PDA@BN-TiO_2_

**Figure 4:**
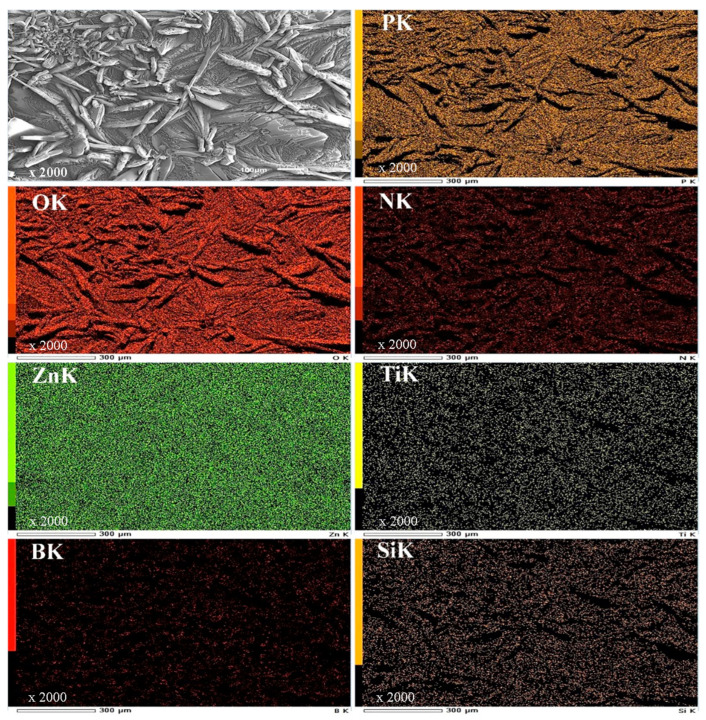
Mapping the chemical composition of a PBT-0.3 sample using EDS. Scale bars in all images are 300 μm.

Compositional analysis of the phosphate-silane layer on the PBT-0.3 specimen surface was characterized by a 20.0 kV energy-dispersive spectrometer (EDS) mapping analysis. Figure 4 depicts the phosphate-silane-coated sample’s elemental distribution and surface shape. The EDS mapping shows that the O and P elements represent the phosphate salts. Large crystals and spaces inside the phosphate sample reflect the phosphate crystals with varying appearances because P and O elements exist consistently across the entire substrate area. This results in both massive crystals and voids inside the phosphate sample. Fe elements were detected in the EDS map, originating from the P-Fe-Zn crystals, the steel substrate, or the phosphophylite Zn_2_Fe(PO_4_)_2_·4H_2_O. The electron beam may penetrate the phosphate coating and reveal a greater concentration of Fe elements because of the coating’s permeability. The phosphate crystals in the phosphating solution were shown to have a higher concentration of Zn elements and Zn^2+^ ions than at the gaps when mapped using EDS. Phosphate-silane coatings were also dense because elemental mappings for B, Si, N, and Ti were concentrated there. Phosphate-silane crystals, as observed in FE-SEM in Figure 3d, may be co-precipitated by the PDA@BN-TiO_2_.

### 3.4. Coating Mass of PDA@BN-TiO_2_ Phosphate Coating

**Figure 5:**
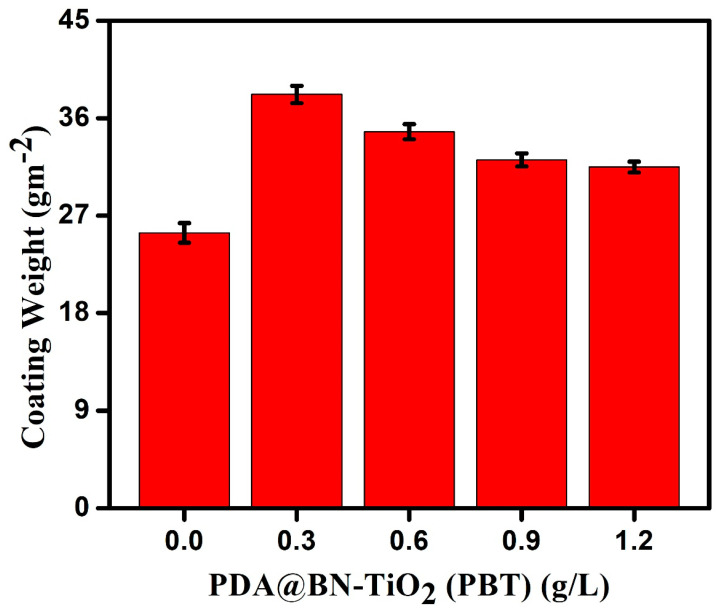
Weight of phosphate coatings versus PDA@BN-TiO_2_ concentration in the phosphating bath.

Steel specimens were submerged in a phosphating bath with different concentrations of PDA@BN-TiO_2_ (0.3 g/L to 1.2 g/L) for 20 min at 40 °C, and the integrity and thickness of the phosphate-silane coatings were assessed by the mass per unit area (MPA). The MPA can be measured by chemically removing the phosphate coatings and calculating the weight loss. It was observed that the MPA was significantly improved by adding PDA@BN/TiO_2_ nanohybrids into the zinc phosphating bath, up to 38.2 g/m^2^, as shown in Figure 5. The MPA of PBT-0 coatings was 25.4 g/m^2^. In contrast, the values of PBT-0.9 and PBT-1.2 decreased to 32.14 g/m^2^ and 31.49 g/m^2^ respectively. This observation resulted in the rapid formation of phosphate crystallization due to the PDA@BN-TiO_2_ nanohybrids.

Consequently, PBT nanohybrids could be eco-friendly catalysts for manufacturing phosphate-silane composite coatings. However, a decrease in coating weight was seen with increasing concentration of PBT, as shown in Figure 5. All measurements were performed three times, and average values were used. Hence, it can be concluded that the PDA@BN-TiO_2_ nanohybrids are effective non-chemical accelerators for fabricating wide-scale phosphate-silane coatings at low temperatures.

We have concluded that PBT-0.3 is ideal for creating a phosphate-silane coating, as the two factors—coating thickness and weight—are generally proportional, with an approximate ratio of 1 μm to 1.5–2 g^−2^ [1]. The research thus indicates that a more substantial and reliable phosphate-silane coating can be produced by integrating PDA@BN-TiO_2_ nanohybrids into the zinc-phosphating bath at a lower temperature than unmodified coatings.

### 3.5. XRD Patterns of PDA@BN-TiO_2_

**Figure 6:**
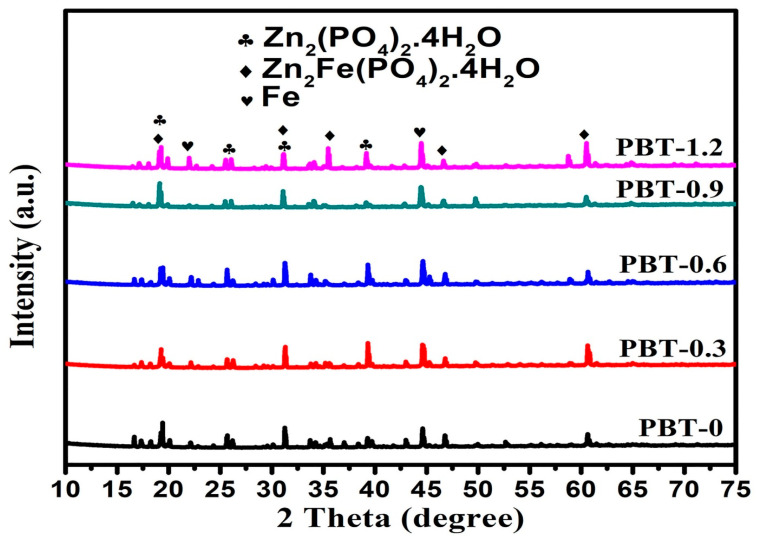
X-ray diffraction patterns of PBT (0.3–1.2 g/L) coatings made of phosphate and silane at different strengths.

The phase composition of PBT-coated steel specimens is characterized by XRD analysis (Figure 6). It is evident from the XRD pattern that the steel specimen has developed Zn_3_(PO_4_)_2_·4H_2_O (hopeite, JCPD file #37- 0465) and Zn_2_Fe(PO_4_)_2_·4H_2_O (phosphophylite, JCPD file #29-1427). The crystal plane (002) of Zn_3_(PO_4_)_2_·4H_2_O exhibits a peak at 19.4°. However, incorporating polydopamine boron nitride-titanium dioxide slightly enhanced the intensities of Zn_3_(PO_4_)_2_·4H_2_O. The addition of polydopamine-modified boron nitride-titanium dioxide (PBT) did not alter the phosphate crystal structure of the coatings, as seen by the XRD pattern, which reveals no change in peak position. Furthermore, the peak intensity of Zn_3_(PO_4_)_2_·4H_2_O increased with an increase in PBT content, signifying an acceleration of the formation of Zn_3_(PO_4_)_2_·4H_2_O, enhancing the preferential growth of the phosphate crystals without changing the coating’s phase composition. Additionally, the progress rate of hopeite was higher than phosphophyllite when in a PBT-containing phosphating bath. The distinctive peaks of BNs were identified at 26.5, 41.5, and 50.1°, and the characteristic peaks of TiO_2_ were observed at 25.31°, 38.01°, and 48.02°, respectively, in the PDA@BN/TiO_2_-incorporated samples. These findings provide proof of the presence of PDA@BN/TiO_2_ in the resulting coating.

### 3.6. XPS Analysis of PDA@BN-TiO_2_ Phosphate-Silane Coatings

**Figure 7:**
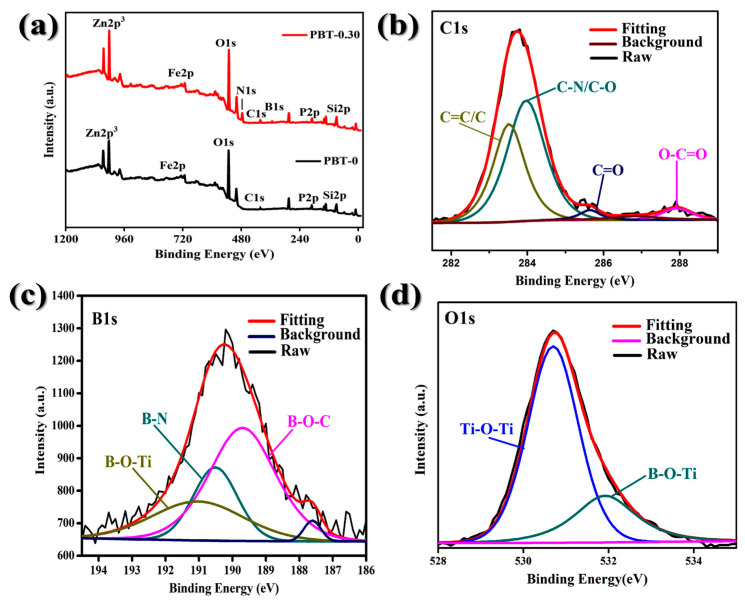
(**a**) XPS survey scan of PBT Phosphate coating. (**b**) C1s, (**c**) B1s, (**d**) O1s.

**Figure 8:**
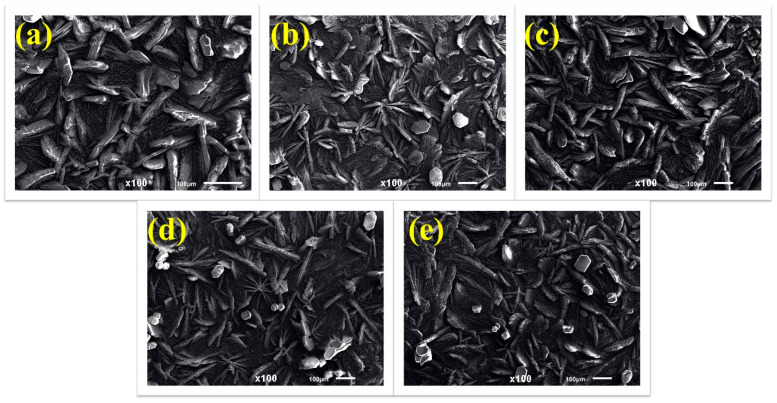
SEM pictures of phosphate-silane coatings from the phosphating bath with different PBT concentrations (**a**–**e**).

After phosphate-silane treatment, the thickness and surface smoothness of the specimen both improved. Analysis of element-specific binding energies via X-ray photoemission spectroscopy (XPS) is used to conduct an in-depth analysis of the phosphate-silane-coating. XPS study was conducted to determine the elements present and the chemical nature of the PBT-incorporated phosphate-silane coating. Figure 7a represents the Spectral survey X-ray spectroscopy of the PBT-0.3 and untreated (PBT-0) samples. The analyses showed that the reaction byproducts of phosphate coating consisted of iron, oxygen, Phosphorus, Zinc, and Carbon elements. On the other hand, the phosphate-silane layer shows abundant Carbon, Oxygen, and Silicon elements, according to the electron binding energy. Silane is composed of three key elements—carbon (C), silicon (Si), and oxygen (O)—represented by its chemical formula “Y(CH_2_)_3_Si(CH_3_)_3_”. Here, Y stands for “(CH_2_OCHCH_2_O)”. Analysis of the elements on the phosphate-silane-coated surface of the steel substrates indicates a high intensity, indicating that a silane coating has been successfully formed (Figure 8a–e) [1,34]. The XPS spectra of PBT-0 and PBT-0.3 showed peak features of Carbon (C1s), Boron (B1s), and Nitrogen (N1s) elements, respectively, as observed in Figure 7a. The increased strength of the Carbon and Boron in PBT-0.3 is associated with the introduction of dopamine-based PDA@BN-TiO_2_ nanohybrids. A complete XPS survey of the specific electron binding energies for various phosphate-silane coating elements was conducted to gain insight into the resulting reaction products of the phosphate-silane-coated steel specimens X [35,36,37]. Figure 7b provided a depiction of the XPS spectra of C1s, with four peaks at 283.8 eV, 284.5 eV, 285.5 eV, and 286.5 eV, which correspond to CeSi, “C=C/C,” CeN and “C–N/C–O” entities respectively [1]. The supplementary peak of the C–O entity signifies the presence of the polydopamine film over the BN/TiO_2_ nanohybrids. Figure 7c represents B1s spectra; the peaks at 190.78 eV, 190 eV, and 191.4 eV verify the development of B–O–C bands, BeN bands, and B–O–Ti bands, respectively [31]. Figure 7d illustrates the O1s spectra at 529.7 eV and 529.1 eV attributed to Ti–O–Ti bands and B–O–Ti bands, respectively [38,39]. The XPS data indicates that the PDA coating of h-BN@TiO_2_ caused an increase in the C content of the laminate surface while also introducing an NH_2_ group. The spectra showed firm peaks of both B and O, attributed to the TiO_2_ bonded to the h-BN. These interactions are linked to the phosphate-silane and PDA@BN-TiO_2_ nanohybrid particle layers. The improved binding strength between the PBT-coated samples and the adhesive may be due to the silanol bonds that form during hydrolysis and the cyclic opening of the silanes during the phosphating procedure. This reaction increases the adhesive-phosphate coating intermolecular interaction and provides a sealing effect for the micro pores. As a result, these bonds make it more difficult to differentiate between the two surfaces and help enhance their bond strength.

### 3.7. SEM Characterization

An investigation of the microstructural changes during the phosphating process was conducted using a scanning electron microscope (SEM), which allowed for assessing the phosphating mechanism and discussing the observed changes and the implications for the material. SEM images in Figure 8a–e demonstrate the observed features of PBT-0, PBT-0.3, PBT-0.6, PBT-0.9, and PBT-1.2 during the phosphating procedure. Figure 8 depicts the progressive formation of phosphate-silane crystals, and incorporating PDA@BN-TiO_2_ accelerates the growth rate. PBT-0.3 sample crystals exhibited the highest rate of development proportionally. PBT-0 does not form crystals, even when the phosphating time increases, resulting in a thin layer of phosphate particles covering the base material. Incorporating PDA@BN-TiO_2_ into the phosphating bath could promote crystallization and induce the precipitation of phosphate crystals under short treatment times of 20 min, as shown in Figure 8. Figure 8b demonstrates the PBT-0.3 sample after phosphating, with the surface entirely covered with small phosphate crystals.

In contrast, the PBT-0 sample in Figure 8a is not entirely coated, leaving most of it uncoated and exposed. The PBT-0.3 specimen Figure 8b obtained the most homogeneous phosphate-silane coating crystal structure. As the concentration of PDA@BN-TiO_2_ nanohybrid particles increases, the formation of finer crystals on the steel surface is observed, bridging the space between the larger crystals of PBT-0.3. Micropores in the phosphate coating are also filled by the silane and steel substrate interaction, as illustrated in Figure 8 for PBT-0.3 and PBT-0.6 samples, which resulted in a homogeneous and evenly dispersed silane layer over the phosphate-silane coating Figure 8b,c.

In addition, it is observed that after the phosphating process, PBT-0.6 and PBT-0.9 include larger phosphate crystals that differ from PBT-0 and PBT-1.2. Here it demonstrates that a thicker and more sensitive phosphate coating is not produced by adding additional PDA@BN-TiO_2_. After phosphating, PBT-0.9 and PBT-1.2 have a less compact structure because the steel substrate has been exposed in some areas.

PDA@BN-TiO_2_ nanohybrid particles were proposed as the catalysts for the deposition of phosphate crystals after they were shown to be deposited with PBT-0.30 in FESEM images, similar to GO sheets, where phosphate crystals develop. PBT-0.3 and PBT-0.6 also had a unique PDA@BN-TiO_2_ content, which resulted in a more significant proportion of PDA@BN-TiO_2_ wrapping the phosphate crystals. PDA@BN-TiO_2_, such as GO nanosheets, favored development in various orientations, changing the overall pattern of phosphate crystals and giving rise to new types of phosphate crystals. Due to this growth, crystals of phosphate-silane coating are strip-shaped. Based on the above analysis, PDA@BN-TiO_2_ could be advantageous and alter crystallization reorganization kinetics.

### 3.8. Phosphating Mechanism

The fabrication of a phosphate-silane coating requires a complex combination of chemical and electrochemical steps, which involves connecting both micro anodic and cathodic terminals [1]. Furthermore, the phosphating process depends on various factors, such as the interaction between a silane and a metallic specimen and the silane hydrolysis. Additionally, immersion of metals into the phosphating bath triggers a series of chemical reactions, which depend on the catalytic reactions of accelerators and oxidation-reduction reactions [40]. Due to the electrochemical degradation of steel in acidic conditions, dipping the sample into a phosphating bath causes an acidic reaction that produces iron (II) ions (Fe^2+^) and releases hydrogen gas (H_2_), which is an anode reaction in the phosphating procedure.

(1) $$Fe+2H^{+}\to {Fe}^{2+}+H_{2}↑$$

The reaction between the base metal and the phosphating solution produces hydrogen (H_2_), which is quickly taken up by the free phosphoric acid, resulting in a rise in pH.

(2) $$H_{3}{PO}_{4}\to H_{2}{PO}_{4}^{-}+H^{+}\to {HPO}_{4}^{2-}+{2H}^{+}\to {PO}_{4}^{3-}+{3H}^{+}$$

When the pH rises, phosphate coating forms on the steel sample, which causes the amorphous salts to change from a soluble primary form to an insoluble tertiary form.

(3) $$2{PO}_{4}^{3-}+{3Fe}^{2+}+{8H}_{2}O\to {Fe}_{3}{\left({PO}_{4}\right)}_{2}\cdot {8H}_{2}O↓$$

(4) $${2Zn}^{2+}+{Fe}^{2+}+2{PO}_{4}^{3-}+{4H}_{2}O\to {Zn}_{2}Fe{\left({PO}_{4}\right)}_{2}\cdot {4H}_{2}O↓$$

(5) $${3Zn}^{2+}+2{PO}_{4}^{3-}+{4H}_{2}O\to {Zn}_{2}Fe{\left({PO}_{4}\right)}_{2}\cdot {4H}_{2}O↓$$

The anodic and cathodic electrodes influence the rate of the entire reaction and will accelerate as the two come closer. The silanol functional groups and high specific surface area of the PDA@BN-TiO_2_ nanohybrid particles could allow particles to adhere to the surface of the steel. Therefore, the phosphating solution forms hydrogen bonds with the silanol functional groups via their -OH groups, initiating the ring-opening reaction, which ultimately results in the formation of a covalent linkage between the substrate and the solution, with at least one silicon bond forming between each silanol group and the sample surface. Because of the self-polymerization phenomenon, the silanol groups in the phosphating solution would react with the considerably more active -OH groups in the polydopamine residue, producing a layer over the steel surface [1]. Silane KH560 hydrolysis reactions with Fe samples are shown schematically as [1]:

(6) $$Y{\left({CH}_{2}\right)}_{3}Si{\left(CH_{3}\right)}_{3}\to Y{\left({CH}_{2}\right)}_{3}Si{\left(CH_{3}\right)}_{3}+H_{2}O$$

(7) $$Fe{\left(OH\right)}_{2}+Y{\left({CH}_{2}\right)}_{3}Si{\left(CH_{3}\right)}_{3}\to OHFe-O-Si{\left(OH\right)}_{2}Y{\left(CH_{2}\right)}_{3}+H_{2}O$$

(8) $$Y{\left({CH}_{2}\right)}_{3}Si{\left(CH_{3}\right)}_{3}+Y{\left({CH}_{2}\right)}_{3}Si{\left(CH_{3}\right)}_{3}\to Y{\left({CH}_{2}\right)}_{3}Si-O-SiY{\left({CH}_{2}\right)}_{3}+H_{2}O$$

The hydrolysis of silane KH560 produces silanol groups (Si-OH) in the silane solution (Equation (6)). Hydroxyl groups in phosphate coatings would interact with the silanol groups resulting in a chemical reaction to form a strong Si-metal bond (Equation (7)). In contrast, the remaining silanol groups would condense to form Si-O-Si bonds (Equation (8)), resulting in a 3D network of highly cross-linked silanol chains. Poly (dopamine)modified BN-TiO_2_ nanohybrid particles, which act as a sedimentary bed and collect dissociative ions of Fe^2+^ and Zn^2+^, speed up the sedimentation process similar to that of graphene oxide —at the same time, enhancing the phosphating process and precipitating the development of insoluble phosphate crystals when untreated. Moreover, PDA@BN-TiO_2_ nanohybrid acts as a nucleation position, leading to a dense and more refined phosphate layer upon the steel surface.

### 3.9. Corrosion Behavior

#### 3.9.1. Potentiodynamic Polarization Characterization

**Figure 9:**
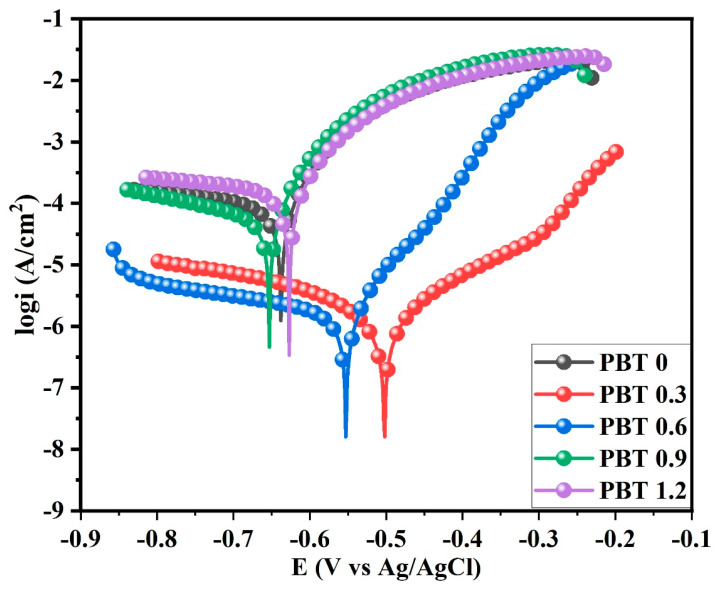
Tafel scan curves of PBT phosphate coating steel specimens.

The corrosion performance of the PBT-phosphate-coated steel samples was evaluated through electrochemical analysis and potentiodynamic polarization using a three-electrode system. The polarization curve results of specimens coated with varying amounts of PDA@BN-TiO_2_ nanohybrid particles were collected and represented in Table 3. It was observed that adding PDA@BN-TiO_2_ nanohybrid particles to the phosphating bath positively affected the corrosion resistance of steel samples, as evident from the polarization curves (Figure 9). The analysis of the thermodynamic stability of the system in a corrosive medium shows that the corrosion potential of the untreated samples is shifted to a more positive potential after applying an anti-corrosive film. Table 3 indicates that the corrosion potential (E_corr_) of the phosphate specimen treated with PDA@BN-TiO_2_ nanohybrid particles is more positive than that of the untreated samples. This shift indicates that the PDA@BN-TiO_2_ nanohybrid coating effectively provides increased corrosion protection. Incorporating PDA@BN-TiO_2_ nanohybrid particles into the PBT coatings had a noticeable effect on the corrosion current density (i_Corr_), manifesting as a lower corrosion current density than the untreated (PBT-0) coating. The most significant shift towards a more positive Ecorr was present in the PBT-0.3 specimen, indicating highly increased corrosion resistance from the addition of these nanohybrid particles. By introducing PDA@BN-TiO_2_ nanohybrid particles, results were observed that the corrosion current density (i_Corr_) had been decreased by at least one order of magnitude. This value was determined by the observed minimum value of 1.63 × 10^−6^ A/cm^2^, indicating a highly increased corrosion resistance within the PBT coating. The addition of PDA@BN-TiO_2_ nanohybrid particles significantly decreased the corrosion rate of stainless steel. The highest corrosion rate of steel samples was observed for the PBT-0. Although the concentration of PDA@BN-TiO_2_ nanohybrid particles increased, the corrosion rate decreased to the lowest point of 8.47 mills per year for the PBT-0.6. This reduction was of one order of magnitude compared to the PBT-0, indicating the effectiveness of PDA@BN-TiO_2_ nanoparticles in forming a resistant coating on the surface of stainless steel, which protects it from corrosion.

Phosphate coatings with greater density and weight achieved superior corrosion resistance qualities because they provided stronger barrier protection against corrosive electrolytic attacks [11]. By examining the SEM images from Figure 7 and the coating weight results from Figure 5, it can be seen that phosphate coatings with a higher density and higher weight exhibited better corrosion resistance qualities. The sample PBT-0.3 achieved the highest degree of corrosion resistance with the highest coating weight and densest coating compactness, which was one order of magnitude greater than the untreated substrate (PBT-0). PBT-0.6 had a lower weight and less dense coating, showing relatively poorer corrosion resistance but still a significant upgrade compared to the pure sample PBT-0. The resilience and ability to resist corrosion of PBT-1.2 were comparable to that of PBT-0.9, with a slight but measurable increase in corrosion protection.

Each phosphate-coated steel sample’s protection efficiency (η) was computed using Equation (1), where $i_{corr}^{0}$ and i_Corr_ is the corrosion current densities of untreated and treated samples with PDA@BN/TiO_2_, respectively. Among all the samples, PBT-0.30 exhibited increased protection efficiency (η). Additionally, Equation (2) was utilized to determine the corrosion rate of each sample, where E, K, and ρ are the steel’s equivalent, equivalent coefficient, and density, respectively [41]. The PBT-0 (untreated) displayed the highest corrosion rate, which decreased with the addition of polydopamine-modified h-BN/TiO_2_. However, with the increased concentration of PDA@BN/TiO_2_, the corrosion rate decreased to a minimum of 4.07 × 10^1^ mills per year for PBT-1.2, one order of magnitude smaller than that of PBT-0.

(9) $$\eta =\frac{\left[\left(i_{corr}^{0}-i_{corr}\right)\right]}{i_{corr}^{0}}\times 100$$

(10) $$CR=\frac{K\times i_{corr}}{\rho \times E}$$

More compact and thicker phosphate coatings provide better protection against electrolytic corrosion, improving corrosion resistance. In our coating tests, SEM analyses in Figure 8 and coating weight tests in Figure 5 indicate that the sample PBT-0.3 displayed the most effective corrosion resistance, with a substantial coating weight and the densest coating compactness, one order of magnitude higher than the untreated substrate. However, the phosphate-coated sample PBT-0.6 showed slightly low coating weight and compactness compared to PBT-0.9, representing less effective corrosion protection but better than the untreated sample MBN-0. The compactness and corrosion resistance of PBT-1.2 were almost similar to that of PBT-0.

The results of this study suggest that incorporating PDA@BN-TiO_2_ nanohybrid particles into phosphate-silane-coated steel can provide an improved corrosion barrier and also helps to limit the penetration and propagation of corrosive ions to the steel surface. This approach offers an effective way to protect steel from corrosion.

#### 3.9.2. Electrochemical Impedance Spectroscopy (EIS)

**Figure 10:**
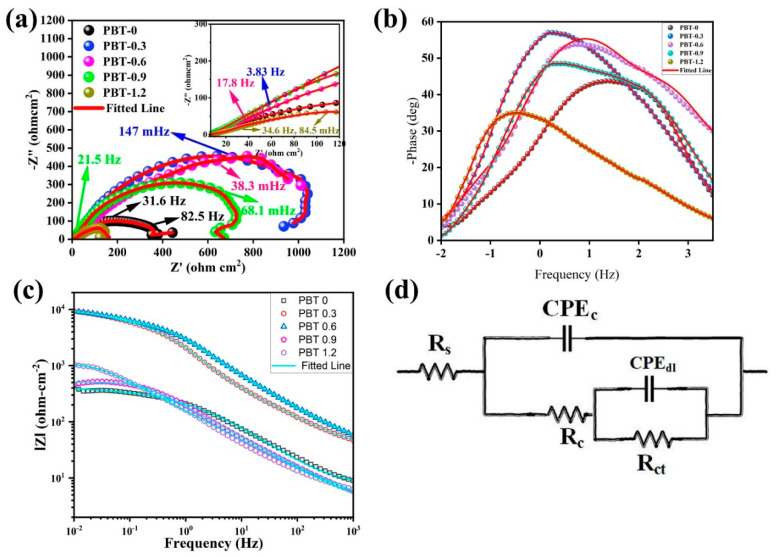
Analysis of Electrochemical Impedance Spectroscopy of Phosphate-Silane Coated Surfaces with Varying PBT Concentrations (**a**) Nyquist plot, (**b**) Phase angle plots, (**c**) Bode plots, (**d**) equivalent circuit diagram.

The electrochemical evaluation of PBT steel samples treated with PDA@BN-TiO_2_ is evaluated employing electrochemical impedance spectroscopy (EIS). EIS data were analyzed by employing the techniques of Nyquist, Bode, and Phase Angle plots. Figure 10 illustrates the electrochemical impedance spectroscopy values for phosphate-silane coatings. The EIS testing experiments were conducted at a standard temperature to obtain linearity measurements, assess the system’s causality, and determine its function’s longevity [42]. The Nyquist plot in Figure 10a illustrates the graphical representation of different levels of PBT concentrations. The Nyquist plots of all the PBT coatings show a semi-circle with varying diameters, demonstrating an increase in the protective performance of the coatings due to their capacitive properties in the early stages of corrosion. When PBT-0.3 was added, the semi-circle diameter increased, as the coating weight tests indicated. Increasing the content of the PDA@BN-TiO_2_ nanohybrid particles beyond a certain point resulted in a decreasing semi-circle diameter. This information suggests that the heaviest coating weight gives a thicker coating thickness. Furthermore, phosphate coatings of greater thickness in a 3.5% NaCl solution also have higher capacitance values because of the insulation properties of these coatings. The development of thicker coatings with a greater level of capacitance is beneficial in terms of chemical protection [43]. The higher the polarization resistance, the larger the semi-circle of the phase angle plot (Figure 10b, which represents the structural integrity of a phosphate-silane-coated substrate [44]. Due to the coating’s interaction with the corrosive constituents at specific frequencies, coating durability is evaluated by phase angle (-θ) [1,7]. The coating containing PDA@BN-TiO_2_ nanohybrid particles resulted in superior phase angles compared to the untreated coating, indicating superior corrosion protection. In addition, they yield higher polarization resistance in the Bode impedance plots (Figure 10c), which demonstrates the strong physical adherence of the coating to the substrate and indicates its excellent physical integrity. The fine grain size of PDA@BN-TiO_2_, which promoted the mechanical bonding of phosphates to the metal substrates, is most likely responsible for the increase in polarization resistance. Impedance can be expressed in terms of a complex number characterized by a real part, magnitude (Z’), and an imaginary part, phase angle (Z”) in Nyquist and Bode plots which are used to analyze Electrochemical Impedance Spectroscopy (EIS) data. The Nyquist plot of zinc phosphate-silane coatings with PDA@BN-TiO_2_ nanohybrid particles incorporated into the phosphating bath demonstrates increased anti-corrosion performance, with a higher magnitude displayed by the Bode Plot and a greater radius of the capacitive loop in the Nyquist plot for the PT-0.3 coating compared to the PBT-0.6. It indicates that the Zn-Phosphate/silane coating with PDA@BN-TiO_2_ nanohybrid particles has more durable protection against corrosion.

The experimental results show that PDA@BN-TiO_2_ nanohybrids added to phosphate-silane-coated steels exhibited excellent corrosion-inhibiting qualities. The analysis of the data is summarized in Table 4. The EEC circuit with a model of R(Q(R(QR))) was developed for further examination of EIS measurements. This five-component model is represented in Figure 10 and provides an analytical approach to investigating the electrical characteristics of organic devices. It enables a better understanding of EIS spectra and serves as a tool to analyze the impedance properties of organic materials [41]. This circuit comprises a solution resistor (Rs) and a charge transfer resistor (Rct). The other circuit components are the phase element, the coating capacitance (CPEc), and the ionic current equivalent resistance through the coating (Rc). The final section in the equivalent circuit of an electrode symbolizes the double-layer capacitance (CPEdl) and the parallel charge transfer resistance (Rct). The capacitance equivalent is given by Z = [Y(jω)^n^]^−1^, where Y represents the admittance as a function of angular frequency (ω). Here, Z denotes the impedance of CPE, n, Y is the coefficient ranging from 0–1, j is the unreal number, and ω is the angular frequency with the highest imaginary impedance [41].

Table 4 illustrates the electrochemical parameters determined by the circuit model. These parameters demonstrate the system’s dependability and the insignificant influence of solution resistance on measured parameter values. Moreover, no substantial variation in R_s_ can be observed. Higher charge transfer resistance, coating resistance, and coating capacitance values are obtained when PDA@BN-TiO_2_ nanohybrids catalyze the phosphating layer. Enhanced weight and density of PBT-phosphate-silane coating offer improved barrier protection for transporting corrosive electrolytes [21]. The addition of PDA@BN-TiO_2_ nanohybrid particles to the phosphate coating of test specimens results in improved corrosion resistance, as indicated by an increase in the uniformity and density of the coating. The increased PDA@BN-TiO_2_ content also contributes to a deterioration in coating and charge transmission resistance. Results show that “R_ct_” values in PBT specimens significantly increased than in PBT-0 (pure) samples, with the highest increase in the PBT-0.3 specimen. However, the further increase in the PDA@BN-TiO_2_ content causes a decrease in the R_ct_ value.

### 3.10. Copper Sulphate Spot Testing

A spot-testing technique was used to evaluate the corrosion performance of the PBT-incorporated phosphate-silane layer. A drop of copper sulfate (CuSO_4_·6H_2_O) was placed on the sample surface, and the time it took for the color to transform was noted. This testing is based on the process of substitution reaction theory, wherein copper ions (Cu^2+^) replace the iron found in the steel specimen [1,45]. When a copper sulfate (CuSO_4_·6H_2_O) solution is applied to a phosphate-coated steel piece, the copper ions react with the iron of the specimen below the phosphate-silane layer, enabled by the permeability of the protective film. The color of the solution droplet will dissipate after the displacement reaction is complete; however, the corrosion of the sample continues. The amount of time required for the color to change depends on the thickness of the phosphate layer.

**Figure 11:**
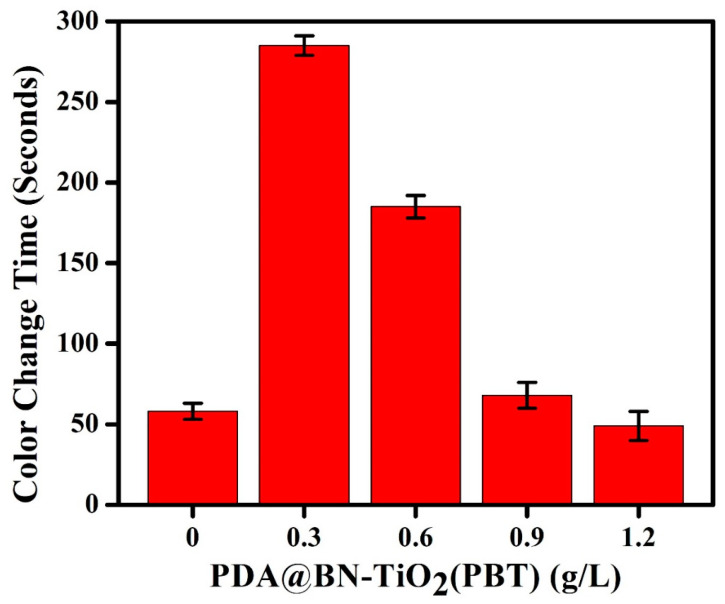
Timing Effects of Copper Sulfate Testing on Phosphate-Silane incorporated PDA@BN-TiO_2_ with mean values.

Figure 11 displays the timing effects of copper sulfate testing on phosphate-silane-incorporated PDA@BN-TiO_2_ nanohybrid particles with mean values. At the lowest concentration (PBT-0), the color changed in 60 s; at higher concentrations, the time increased to 285 s for PBT-0.3. The 0.3 concentration was deemed the ideal amount for optimum anti-corrosive efficiency, as demonstrated when testing coating weight, performing SEM observations, and analyzing Tafel and EIS characterization curves. As a result, PBT-0.3 was identified as the most successful phosphate-silane coating.

## 4. Conclusions

In this study, PDA@BN-TiO_2_ nanohybrid particles were studied for their potential to improve the corrosion protection of a silane-phosphate coating on Q235 steel when subjected to a phosphating solution. By utilizing varying PDA@BN-TiO_2_ (0.3–1.2 g/L) concentrations for the phosphating solution, SEM characterizations and XPS analyses have revealed that the addition of PDA@BN-TiO_2_ nanohybrid particles could form a more dense and less permeable phosphate coating than untreated samples, forming sites for the crystallization of phosphate and the deposition of metal ions, enhancing the phosphate layer on a metal surface. The PDA@BN-TiO_2_-nanohybrid particle incorporated phosphate samples treated with KH560 develop a denser and more refined coating structure., resulting in better corrosion resistance performance in a 3.5% NaCl aqueous solution. A notable enhancement in corrosion resistance was observed by comparing treated PBT steel and untreated PBT coating. Compared with other samples, the PBT-0.3 sample (0.3 g/L of PDA@BN-TiO_2_ nanohybrid particles) demonstrates the most significant coating weight (38.2 g/m^2^) and extraordinary corrosion resistance (1.63 × 10^−6^ A/cm^2^ of iCorr and 285 s for CuSO_4_·5H_2_O testing). Therefore, PDA@BN-TiO_2_ nanohybrid particles could be utilized as a low-temperature phosphating accelerator for superior corrosion resistance.

## Figures and Tables

**Table 1:** Chemical composition of Grade Q235 mild steel.

Element	C	S	Si	P	Mn	Fe
**wt%**	0.22	0.05	0.35	0.05	1.40	Bal.

**Table 2:** Chemical constituents, operational parameters of a zinc-phosphating bath.

Formulations	Concentration	Operating Conditions
H_3_PO_4_ (85%)	3.0 g/L	Temperature (T): 40 °C
KH560 (Silane)	1.50 g/L	Period: 30 min
H_3_PO_3_ (99%)	2.0 g/L	
Zn(H_2_PO_4_)_2_·2H_2_O	50 g/L	
Zn(NO_3_)_2_·6H_2_O	80 g/L	
PDA@h-BN-TiO_2_	0.3–1.2 g/L	

**Table 3:** Polarization Curve Parameters were obtained by adding different content of PBT.

Sample Code	E_Corr_ (V)	*i*_Corr_ (A/cm^2^)	Corrosion Rate (mm/Year)	R_p_	η%
PBT-0	−0.531	1.43 × 10^−4^	2.87 × 10^1^	287	-
PBT-0.3	−0.498	1.63 × 10^−6^	7.46 × 10^1^	2387	98%
PBT-0.6	−0.557	1.85 × 10^−6^	8.47 × 10^1^	2088	97%
PBT-0.9	−0.540	6.21 × 10^−5^	2.85 × 10^1^	1367	56%
PBT-1.2	−0.561	1.65 × 10^−4^	4.07 × 10^1^	985	15%

**Table 4:** Circuit-based electrochemical Values.

Sample	R_s_	CPE_c_	n	R_c_	CPE_dl_	n	R_ct_
PBT-0	6.47	2.42 × 10^−4^	0.55	22.90	9.5 × 10^−4^	0.52	378
PBT-0.3	5.74	1.77 × 10^−5^	0.65	35.56	7.19 × 10^−5^	0.62	935
PBT-0.6	5.34	2.41 × 10^−5^	0.62	34.48	5.07 × 10^−5^	0.60	825
MBN-0.9	3.71	7.57 × 10^−4^	0.58	28.72	4.67 × 10^−4^	0.61	489
PBT-1.2	4.42	3.30 × 10^−4^	0.54	24.53	3.56 × 10^−4^	0.52	359.5

## Data Availability

The data presented in this study are available within the article.
